# Smart Cardiac ICU: Digital Integration, Predictive Analytics, and Perioperative Inflammation

**DOI:** 10.3390/bioengineering13080921

**Published:** 2026-08-14

**Authors:** Leonard Azamfirei, Mihaly Veres, Sanziana Bora, Mirela Cecilia Oiaga, Mihaela Butiulca, Alexandra Elena Lazar, Janos Szederjesi, Bianca Liana Grigorescu

**Affiliations:** 1 Department of Anesthesiology and Intensive Care, George Emil Palade University of Medicine, Pharmacy, Science and Technology of Targu Mures, 540142 Targu Mures, Romania; leonard.azamfirei@umfst.ro (L.A.); mihaela.butiulca@umfst.ro (M.B.); janos.szederjesi@umfst.ro (J.S.); bianca.grigorescu@umfst.ro (B.L.G.); 2 Doctoral School of Medicine and Pharmacy, George Emil Palade University of Medicine, Pharmacy, Science and Technology of Targu Mures, 540139 Targu Mures, Romania; 3 Department of Anesthesiology and Intensive Care Medicine, Emergency County Hospital, 540136 Targu Mures, Romania; 4 Department of Anesthesiology and Intensive Care, Emergency Institute for Cardiovascular Diseases and Transplantation, 540136 Targu Mures, Romania; flamind.sanzi@gmail.com; 5 George Emil Palade University Hospital, 540142 Targu Mures, Romania

**Keywords:** smart ICU, cardiac surgery, cardiopulmonary bypass, predictive analytics, perioperative inflammation, clinical decision support, interoperability

## Abstract

Contemporary intensive care operates in an environment with high-complexity cases, large volumes of information, and vast physiological, biological, and therapeutic data, collected from laboratory results, investigations, and therapies for organ support, as well as from systems that operate in parallel. The lack of interoperability contributes to information overload, alarm fatigue, and delayed decision-making. The Smart ICU concept has been developed to address these limitations by integrating medical devices, information systems, and artificial intelligence into a unified system that allows interoperable data integration and predictive analytics. Aim: The purpose of this article is to provide a narrative review of the Smart ICU concept, with a specific focus on the cardiac intensive care unit. It summarizes Smart ICU architecture, data integration, clinical support, and applicability in monitoring perioperative inflammation in cardiac surgery. We describe the Smart ICU architecture, from data acquisition to storage and analytics, highlighting the differences between Smart ICU, artificial intelligence, and Tele-ICU, and we underline predictive analytics as a supportive tool, as well as its influence on clinical outcomes. Cardiac ICU application: Cardiac ICUs offer a data-dense, temporally well-defined model following cardiac surgery with cardiopulmonary bypass, where data concerning patients’ hemodynamics, perfusion data, and biological and inflammatory markers intertwine. Cardiac Smart ICU models could recognize early signs of hemodynamic compromise and low cardiac output states and identify early indicators of post-cardiac surgery complications. Neutrophil activation and complete blood count-derived indices may be used as dynamic biological data for Smart Cardiac ICU models. Conclusion: The Smart Cardiac ICU may support earlier risk stratification, and therefore earlier diagnostic and therapeutic interventions, but its clinical value requires prospective, multicenter validation. Cardiopulmonary bypass-induced inflammation may offer an ideal setting to integrate physiological, procedural, and immunological data into bedside predictive models.

## 1. Introduction

Contemporary intensive care is operating in an environment with high-complexity cases, large volumes of information, and vast physiological, biological, and therapeutic data. These data arise from multimodal monitoring, laboratory results, and therapies for organ support, such as mechanical ventilation, continuous renal replacement therapy, and extracorporeal membrane oxygenation (ECMO). Real-time integration of this immense amount of data is beyond human capacity, resulting in an increased interest in artificial intelligence-based clinical decision support systems [1].

Conventional intensive care encounters several limitations. First, the overwhelming amount of data leads to decision fatigue and delays prompt therapeutic interventions. The burden of information overload is amplified by the lack of interoperability between the systems that generate those data, operating in parallel, representing a major drawback in critical situations where rapid decision-making is mandatory. Second, alarm fatigue adds to the burden of healthcare workers: 72–99% of alarms are either false or clinically irrelevant [1,2,3]. Because of the abundance of clinical parameters, predictive scores, and continuous monitoring, intensive care units are considered key settings for implementing data-driven medicine.

The Smart ICU concept is showing promising results by integrating medical devices, information systems, Internet of Things technologies, and artificial intelligence into a unified system that allows real-time monitoring of a critical patient, enables risk stratification and prediction, and offers decision support and potentially Tele-ICU-based care [4,5,6]. However, routine clinical implementation remains limited.

Although Smart ICU, digital ICU, Tele-ICU, and AI-assisted critical care are often used as common support systems with many similarities, they describe distinct concepts. A digital ICU focuses on data acquisition and storage, integrated into interoperable systems based on digital health records. Tele-ICU requires infrastructure that allows remote monitoring and intervention from a specialized consultant or experienced physician. AI-assisted monitoring uses machine learning algorithms and models that are capable of generating predictive values based on the collected data, early detection of deterioration, or interpretation of physiological signals. In contrast, the Smart ICU concept represents an integration of these technologies into a unified ecosystem that enhances precision critical care.

A recent systematic review of 1263 studies reported that 74% of artificial intelligence (AI) models are at a technology readiness level (TRL) rating of 4 or below, and only 2% are feasible for clinical integration (TRL ≥ 6). The field is experiencing an annual growth rate of 39% in publication volume but is dominated by retrospective studies [7].

Cardiac surgery provides a suitable model for the Smart ICU. It combines the surgical intervention with hemodynamic, perfusion, biological, and inflammatory data, leading to measurable postoperative outcomes [8].

The aim of this narrative review is to provide a comprehensive overview of current data on the Smart Cardiac ICU, with a focus on the digital integration of perioperative data and predictive analytics for decision-making support within the cardiac surgical intensive care setting. We describe the digital architecture of the Smart ICU, related concepts, and AI tools used for prediction, and discuss the current limitations and future directions of this concept. Unlike previous reviews that focus on isolated AI applications, this review emphasizes the possible benefits of integrating these complementary components into the Smart Cardiac ICU architecture. A dedicated chapter on the post-CPB inflammatory response, used as a representative example, emphasizes the feasibility of AI tools integrated in clinical practice in such a complex field, and how cardiac ICUs represent a key setting for data-driven critical care.

## 2. Methodology

This article is a narrative review of the Smart ICU concept, focusing on the cardiac ICU and aiming to present current evidence and research future directions. Its main purpose is to combine digital infrastructure and interoperability, machine learning-based predictive analytics, tele-critical care, and the inflammatory response to cardiopulmonary bypass into a single clinical setting.

We performed a database search on PubMed/MEDLINE and Web of Science, using the following key-words: “intensive care unit”, “Smart ICU”, “intelligent ICU”, “clinical decision support”, “patient data management system”, “alarm fatigue”, “Internet of Things”, “tele-ICU”, “tele-critical care”, ”machine learning”, “artificial intelligence”, “cardiac surgery”, “cardiopulmonary bypass”, “systemic inflammatory response”. The search included articles published between 1 January 2015 and 30 June 2026.

We included original articles, observational studies, randomized trials, systematic reviews, meta-analyses, and consensus documents published in English or Romanian, with relevance to at least one of the central themes of the review: Smart ICU architecture, IoT and alarm management, artificial intelligence in ICU, tele-ICU and perioperative inflammation in cardiac surgery. We excluded articles focused on non-critical fields and reports with no clinical implementation of the Smart ICU concept, case reports, or animal and in vitro studies. Additional references were identified through manual screening of the bibliographies of the selected articles.

We chose a narrative rather than a systematic design because the aim of this review is conceptual: to collect evidence from clinical integration of this concept and highlight the knowledge gaps in the current literature. Therefore, we did not follow the PRISMA methodology and did not perform a quantitative synthesis.

### 2.1. The Smart ICU Concept and Its Application in the Cardiac ICU

Over the last six decades, continuous monitoring and specific organ support therapies have evolved, and conventional intensive care units now rely on numerous sources that generate clinical and paraclinical data. Major disadvantages include a lack of interoperability between monitors, ventilators, infusion pumps, and laboratory findings [9]. Therefore, the clinician remains the “brain” behind this heterogeneous information, with the high amount of information creating cognitive burden and increasing the risk of errors [10]. Evolving digital technologies—electronic records, computerized prescribing systems, and tele-ICU platforms—focus on limiting this issue so as to reduce diagnostic time and the information overload encountered by clinicians [11,12].

The Smart ICU represents a paradigm shift from a fragmented, reactive model of care to an integrated and predictive ecosystem. Conventional intensive care is evolving through the incorporation of communication technologies, Internet of Things (IoT) networks, artificial intelligence, robotics, and big data analytics, with the purpose of improving real-time monitoring, enabling risk stratification and prediction, and improving diagnostic accuracy. Given the scale and complexity of modern critical care systems, digital integration and standardization allow monitoring and analysis to become an interconnected system, linked through IoT and high-speed network infrastructure [5].

Several related terms are often used interchangeably in the literature; Table 1 distinguishes them to clarify the scope of this review.

Unlike conventional ICUs, smart ICUs integrate and analyze large volumes of data, enabling prediction and decision support. AI models with AUC values that are frequently >0.80 are capable of providing early warning and decision support, but only in the presence of robust integration with EHR; calibration and external validation of these models remain suboptimally reported [13]. Specifically, AI models dedicated to sepsis prediction can anticipate its onset 6–12 h before conventional clinical diagnosis, exceeding the accuracy of established scores such as the SOFA and qSOFA. Similarly, the prediction of acute kidney injury 12–48 h in advance, intraoperative hypotension 15 min in advance, and the optimization of ventilator weaning—with a reduction in the duration of mechanical ventilation of about 21 h—concretely illustrate the transformative potential of AI integration into clinical workflows [14].

The Smart ICU requires three interdependent dimensions: the infrastructural dimension, including medical devices, high-speed networks, and digital platforms; the analytical dimension, including machine learning (ML), Internet of Things (IoT), and predictive algorithms; and the human–organizational dimension, which involves staff training and focuses on algorithm-assisted decision-making [5,10,11,12,13,14,15,16].

Digital integration of data carries significant benefits. Recent data from the literature show that implementation of a patient data management system (PDMS) is associated with a reduction in ICU length of stay, lower mortality rates, and lower rates of nosocomial infections, including the elimination of *Acinetobacter* spp. infections [11]. Electronic prescribing has been shown to facilitate earlier de-escalation of antimicrobial therapy and a reduction in antimicrobial resistance [11,17]. Among other benefits of digitalization, it has been shown that by reducing documentation errors and optimizing resource allocation, Smart ICU enhances patient outcomes and clinical workflow. Telemedicine has become a component of the Smart ICU concept, developed and accelerated by the COVID-19 pandemic, where tele-rounds, telemonitoring, and teleconsultations became a necessity [18].

Cardiac surgery can represent a key setting for implementing these tools in clinical practice. Starting from the surgical procedure as a well-defined procedural event with a defined inflammatory trigger, such as cardiopulmonary bypass, allowing dynamic monitoring of postoperative data may predict postoperative outcomes. These features underline the feasibility of using the cardiac ICU as a demonstrative model.

### 2.2. Digital Integration and Interoperability

The Smart ICU uses a layered digital architecture that combines signals with clinical decisions.

The data acquisition layer collects data from specific monitoring systems—monitors, infusion pumps, ventilators, ECMO systems, point-of-care analyzers—and enables synchronization with EHR data to obtain information about trends and changes in the patient’s clinical course [19,20].

The interoperability layer is based on specific universal systems, like HL7, DICOM, and FHIR, which facilitate synchronization of collected data [21,22].

The data storage layer enables storage and transmission of patient information, including Patient Data Management Systems (PDMS), improving data completeness and enabling trend analysis over time [11,22].

The analytic layer acts as the Smart ICU’s “brain” by integrating clinical decision support systems (CDSS), AI algorithms, and predictive models to optimize the decision-making process and anticipate specific complications, such as acute kidney injury and delirium [19,23,24,25].

The clinical interface layer converts data into specific information targeting specific healthcare workers, such as physicians, nurses, and pharmacists. A specific command center provides guidance and coordination, leading to better adherence to clinical protocols that are currently in use [25,26].

The governance and cybersecurity layer manages access and security policies, limiting the risk of bias and ensuring compliance with ethical and legal requirements.

Interoperability relies on standardized data and clinical terminology. Health Level Seven (HL7) and Fast Healthcare Interoperability Resources (FHIR) are examples of programming interfaces that facilitate data exchange. LOINC provides universal codes, SNOMED CT offers clinical terminology, while platforms like DICOM facilitate integration of medical imaging by storing and sharing the above. The Observational Medical Outcomes Partnership (OMOP) Common Data Model supports the harmonization of multicenter datasets for clinical research and AI development. Combined, these standards are essential for interoperable, secure ICU systems.

A recently published Romanian study concluded that automated PDMS documentation was associated with a reduction in hospital length of stay and improved mortality rates [11]. AI tools using multimodal data have shown better outcomes compared to conventional triage tools for prediction of specific infections, such as surgical site infections and urinary tract infections, but validation of these models and clinical feasibility still remain a challenge [27,28,29].

In the cardiac ICU, interoperability becomes particularly important. Hemodynamic data can be continuously monitored using pulmonary artery catheters, which provide measurements of cardiac output, pulmonary artery pressures, and mixed venous oxygen saturation. Echocardiography provides data regarding ventricular function, valvular pathologies, and responsiveness to fluid administration, thereby guiding fluid therapy. These variables could be integrated into a machine learning model, enabling predictive models to identify early hemodynamic changes and guide postoperative management.

Mechanically circulatory support devices further illustrate the value of interoperable Smart ICUs. Temporary mechanical circulatory support devices, including veno-arterial extracorporeal membrane oxygenation (VA-ECMO), ventricular assist devices (VADs), and percutaneous circulatory support systems, generate large amounts of physiological data that are continuously monitored. Both hemodynamic and device-related parameters, more specifically pump flow, pressures, and anticoagulation variables, can be integrated into a predictive model that has the potential to enhance detection of circuit complications, evaluate and predict weaning success, and detect adverse events earlier.

Table 2 summarizes the main data sources of the Smart Cardiac ICU and their potential predictive value.

### 2.3. Predictive Analytics and Clinical Decision Support

Predictive analytics can support clinical decisions, aiding digitalization, reshaping clinical care, and positioning ICU as a leading field. Continuous monitoring, laboratory data, and therapeutic interventions generate large volumes of data, exceeding human cognitive capacity [30,31]. Machine learning (ML) and deep learning can recognize patterns and predict severity and early deterioration. Models such as recurrent neural networks like long short-term memory (RNN-LSTM) can collect data in a dynamic manner, reducing the use of static scores and intermittent assessments [32,33]. AI applications in ICU care can be organized by outcomes.

Most current prediction models use supervised learning for predicting mortality, acute kidney injury, prolonged mechanical ventilation, or other specific outcomes. Unsupervised learning uses hidden patterns and enables identification of patient phenotypes, whereas reinforcement learning requires an AI model to undergo trial-and-error processes in order to optimize treatment decisions; however, its use remains limited in clinical practice. Multimodal AI combines signals, laboratory data, imaging, and electronic health records with the purpose of improving predictive performance.

An ICU patient dynamic generates a vast amount of data. By integrating these multimodal data, clinical decision support systems (CDSSs) can generate recommendations in real time. In a Smart Cardiac ICU, these systems can facilitate vasopressor use through early detection of hemodynamic changes; assess fluid responsiveness by correlating hemodynamic data with echocardiographic data; augment ventilation management through early detection of respiratory deterioration or assess ventilator weaning trials; and have the potential to enhance early detection of acute kidney injury and delirium. Multimodal data integration showed promising results in sepsis prediction through the integration of laboratory data; respectively, in the postoperative setting, integration of laboratory and thromboelastographic data with coagulation parameters may improve the prediction of postoperative bleeding. Therefore, AI-based technologies have the potential to facilitate timely intervention and individualize patient management. These examples illustrate how the Smart Cardiac ICU can transform predictive models into bedside decision support systems designed to augment clinical judgment rather than replace it.

Regarding mortality, a systematic review compared commonly used scores in the ICUs, such as the APACHE, SOFA, and SAPS II scores, with XGBoost and random forest machine learning algorithms, using data from the first 24 h after admission. ML models demonstrated better results compared with conventional scoring in predicting sepsis mortality and length of stay in the ICU [34,35,36].

Regarding length of stay—a meta-analysis of 33 studies reported a pooled AUROC of 0.9005 for the prediction of ICU or hospital length of stay [36].

Assessments of perioperative risk using AI tools have also shown promising results in predicting severe complications, outperforming conventional risk scores. The MySurgeryRisk and POTTER systems have shown promising results regarding morbidity and mortality in over 380,000 patients [37,38]. In a large cohort of over 220,000 patients, XGBoost achieved better performance compared to EuroSCORE II in predicting in-hospital mortality [39].

Regarding ventilation and pulmonary complications, boosting models may detect postoperative pneumonia earlier and may support predictions for requiring mechanical ventilation or successful ventilation weaning. ML may also predict prolonged ventilation in cardiac ICUs [40].

Among other syndromes encountered in the postoperative period, acute kidney injury can be detected earlier using multimodal models incorporating preoperative and intraoperative data [38].

Predictive analytics using neuromonitoring and specific biomarkers can be used to predict postoperative delirium by detecting early alterations preceding cognitive dysfunction [41].

Considering the dynamics of several complications encountered in cardiac ICUs, AI-assisted prediction could be particularly useful. Postcardiotomy shock, for example, is characterized by a persistent low cardiac output and driven by cardiopulmonary bypass-related inflammation, vasoplegia, myocardial dysfunction, and pulmonary hypertension [42], and could be identified earlier by using machine learning models based on perioperative variables, continuous hemodynamic monitoring, and vasopressor requirements. Similarly, the risk of postoperative atrial fibrillation and graft dysfunction after coronary artery bypass grafting could be identified earlier through continuous multimodal data integration. This illustrates how the concept of Smart Cardiac ICU can extend beyond general ICU digitalization by focusing on specific aspects of cardiovascular critical care.

Perioperative inflammation will be discussed in the following sections.

Challenges encountered by predictive models are the result of incomplete and irregularly collected data. Model performance depends on preprocessing capacity, handling missing values, and clear definitions of the intended outcomes [43,44]. Explainable AI tools, such as SHAP and LIME, are used to interpret machine learning models’ predictions and improve predictive accuracy [44,45]. Another significant advantage of explainable AI lies in its transparency, which can provide a rationale for generated predictions and recommendations, thereby increasing physician trust. These advantages are mandatory for safe integration into clinical practice, supporting clinical decision-making and improving regulatory acceptance.

Foundation models and large language models (LLMs) may facilitate clinical information generation by extracting and summarizing data from electronic health records; however, their use remains limited by the lack of prospective validation.

AI implementation in clinical practice remains limited due to the risk of bias (50%), data heterogeneity, and ethical concerns. To overcome these challenges, Smart ICUs require standardized data with immediate availability to improve their effectiveness and explainable AI frameworks [7,27,46,47]. In this context, cardiac surgery provides an ideal setting for integrating physiological, procedural, and immunological data into bedside predictive models.

To summarize, Table 3 provides an evidence synthesis of current data found in the literature regarding AI models and their clinical applications.

This table summarizes the main limitations of the current literature. Most published AI studies are retrospective, single-center, and lack external validation. These limitations are major sources of bias and limit generalizability. To address these challenges, prospective, multicenter studies with external validation are mandatory before implementation of AI technology into clinical practice.

Table 4 summarizes the comparison between current AI models and conventional scoring systems.

### 2.4. Alarm Management, IoT, and Command Center Models

The Internet of Things (IoT) and the Internet of Medical Things (IoMT) represent the technological foundation of the Smart ICU, enabling the continuous collection of data from different medical devices. One IoT architecture was designed to collect and process approximately 22 TB of medical data per year [26].

Alarm overload remains a significant problem in ICUs, generating up to 152.5 alarms/bed/day, 72–99% of which are false positives [51,52,53], taking up to 35% of nursing time and therefore affecting clinical performance [54,55,56]. AI-based solutions imply filtering strategies and mobile notifications that can improve response times and reduce alarm fatigue [2,53,57]. Wearable devices such as smartwatches, by prioritizing alarms according to urgency, have been reported to reduce unnecessary notifications, increase response rates, and support the concept of the “Silent ICU”—replacing conventional bedside alarms with targeted alerts [2,53,54,58].

Command-center models extend these functions by centralizing monitoring and coordination, expanding access to critical care experts and improving adherence to protocols [26].

### 2.5. Tele-ICU and Distributed Cardiac Critical Care

Telemedicine in the intensive care unit (Tele-ICU) enables real-time remote monitoring and clinical support for patients in separate ICUs. A hub-and-spoke architecture allows a central hub to provide support and clinical guidance to smaller units. Other models have been developed for hybrid approaches and consultative interventions, showing promising results in different healthcare environments.

Recent literature emphasizes that Tele-ICU should be considered an adaptable system to local resources and organizational structures [59]. It may be particularly helpful in units with limited access to intensive care physicians, where remote support can improve the decision-making process and resource utilization, facilitate treatment planning, and shorten diagnostic times, with evidence suggesting benefits for critical care capacity, but no impact on reducing ICU transfers [60].

The TELESCOPE trial, the largest randomized trial evaluating Tele-ICU, assessed the effectiveness of daily multidisciplinary rounds conducted by an intensivist through telemedicine across 30 ICUs, reporting no significant reduction in ICU length of stay or hospital mortality [61]. These results were attributed to the operational model and implementation protocol rather than to a lack of value of tele-critical care in general [58]. Another systematic review including 26 studies reported low mortality rate reductions in ICU settings, highlighting the heterogeneity of data and the lack of standardized methodological and clinical protocols. Future studies should compare different Tele-ICU models to determine which approaches are most effective in specific critical care settings [62].

Nevertheless, Tele-ICU has the potential to become a connectivity platform once certain needs are met, such as international standards for definitions and specific protocols regarding training and implementation of tele-critical care models [63]. In cardiac ICU settings, preoperative echocardiographic parameters have been demonstrated to be independent predictors of the need for inotropic support and length of stay [64]. These findings emphasize the need for structured data collection in order to properly integrate Smart ICU systems into clinical practice and obtain valuable information to support clinical decision-making.

A recent systematic review found that hub-and-spoke and hybrid models provided the most consistent clinical and staffing benefits, with tele-ICU nurses playing a major role in enhancing care quality [65]. The COVID-19 pandemic accelerated the need for and implementation of tele-ICU technologies, emphasizing the benefits of telemonitoring and teleconsultation, leading to a major shift in infrastructure designs [66].

Overall, Tele-ICU has significant potential for expanding access to expertise and standardizing care; however, challenges are still encountered, mostly because of financial and infrastructure barriers [67].

### 2.6. Perioperative Inflammation After Cardiac Surgery

Cardiac surgery is a field with particular pathophysiological mechanisms, with perioperative inflammation secondary to cardiopulmonary bypass (CPB) representing the main trigger of complications that affect patient courses and outcomes. Despite advances in understanding and managing the systemic inflammatory response to CPB, it remains a challenging, multifactorial process associated with significant complications, ranging from mild organ dysfunction to severe multiple organ dysfunction syndrome (MODS), and contributing to an increase in mortality in cardiac ICU settings.

The inflammatory response is triggered by surgical trauma, ischemia–reperfusion injury, blood contact with the CPB circuit, coagulopathy, and microcirculatory dysfunction. The release of inflammatory cytokines leads to an increase in vascular permeability, glycocalyx degradation, and vasoplegia. Depending on the duration of CPB and ischemia time, it may lead to organ injury requiring specific organ-support therapies [68].

#### 2.6.1. Inflammatory Biomarkers

Inflammatory biomarkers have emerged as valuable tools for risk stratification in cardiac surgery. Elevated preoperative C-reactive protein (CRP) levels are associated with an increased risk of major cardiac events and all-cause mortality in cardiac ICUs, while elevated levels in the postoperative period have been shown to be an independent predictive factor for mortality [69]. Among inflammatory markers, IL-6, IL-10, IFN-γ, and IL-2 have been associated with prolonged mechanical ventilation, acute kidney injury, and 30-day mortality [70]. These findings support a dynamic multimarker approach to the assessment of postoperative inflammation.

Neutrophils and markers of their activation have gained particular interest as early indicators of postoperative inflammation. A 2025 study reported that neutrophil activation occurs intraoperatively during the CPB period and can serve as an early marker of the inflammatory response, preceding elevations of CRP and IL-6 levels. The transient immune dysfunction induced by CPB and persisting during the postoperative period has been shown to contribute to postoperative coagulopathy and infectious complications [71].

Complete blood count-derived inflammatory indices, particularly the neutrophil-to-lymphocyte ratio (NLR) and systemic immune-inflammation index (SII), have shown prognostic value in cardiac surgery, specifically for postoperative acute kidney injury, prolonged mechanical ventilation, and atrial fibrillation. As independent risk factors for these complications, elevated NLR and SII are associated with longer ICU stays and increased mortality rates [72,73,74].

The inflammatory response to CPB is caused by several overlapping mechanisms, including cytokine release, complement activation, endothelial dysfunction, and oxidative stress. Secondary to leukocyte activation due to the release of pro-inflammatory markers like IL-6, IL-8, tumor necrosis factor-α (TNF-α), and high-mobility group box 1 (HMGB1), the endothelial glycocalyx is injured. Neutrophil extracellular trap (NET) formation and inflammasome activation are interconnected innate immune mechanisms that further amplify the immune response. This process leads to increased vascular permeability, which is clinically translated into vasoplegia and hemodynamic instability.

#### 2.6.2. Relevance to the Smart Cardiac ICU

Rather than evaluating specific biomarkers in a static manner with isolated measurements, Smart ICU systems can analyze trends in values and integrate them with hemodynamic and other patient-related relevant data, supporting the concept of a dynamic inflammatory profile. The interpretation of specific markers of inflammation requires correlations with multiple variables, such as the procedure type, CPB and ischemia time, vasopressor requirements, patient comorbidities, and hemodynamic status. Artificial intelligence can combine these data and generate predictive scores to identify patients at risk for CPB- and inflammation-related complications [68].

In this context, CPB-induced inflammation can be incorporated into a “smart immune-monitoring” framework that may enable earlier identification of high-risk patients and influence perioperative care, thereby optimizing treatment strategies and patients’ outcomes.

Since current biomarkers alone have limited predictive accuracy, the development of multimodal predictive models represents a promising application of Smart ICU technology [69,70,71].

Beyond inflammatory monitoring, the Smart Cardiac ICU concept may differentiate specific postoperative phenotypes based on cardiovascular-specific data. For example, echocardiographic findings combined with data collected from pulmonary artery catheters, inflammatory biomarkers, and vasopressor therapy could help differentiate postoperative low cardiac output syndrome from vasoplegia, isolated right ventricular failure, or myocardial dysfunction. By characterizing distinct phenotypes, Smart Cardiac ICU technologies may lead to more individualized management in the postoperative period.

## 3. Smart Monitoring of the Inflammatory Response

The heterogeneous nature of the inflammatory response induced by extracorporeal circulation has encouraged the use of artificial intelligence and machine learning (ML) for risk stratification and prediction of postoperative complications. ML algorithms are able to integrate data generated from different devices (infusion pumps, ventilators, monitors, and invasive monitoring systems) simultaneously, generating dynamic, real-time information that can influence clinical decision-making. The ability of ML to identify complex patterns supports the concept of AI-based patient phenotyping, enhancing the early detection of patients at risk for postoperative adverse outcomes.

Early applications of artificial intelligence in cardiac surgery focused on predicting postoperative systemic inflammatory response syndrome (SIRS). ML models, including random forest, combined with explainability methods such as SHAP, have shown good results in predicting and identifying modifiable factors such as anemia and elevated lactate levels as independent risk factors for SIRS [75]. NLR and CRP have been reported as independent predictors of increased mortality associated with severe inflammatory response, and ML models have reportedly predicted higher rates of myocardial dysfunction among patients with higher levels of NLR and CRP [76].

A major advantage of machine learning is its ability to identify distinct inflammatory phenotypes following extracorporeal circulation (ECC). Recent data have stratified patients into different subgroups with specific biomarker profiles related to specific clinical outcomes: α phenotype—older patients with multiple comorbidities, longer hospital stays, and higher mortality; and β and γ phenotypes have more favorable outcomes [77]. This major breakdown in ML findings confirms that the inflammatory response to ECC is heterogeneous, therefore suggesting that AI can improve risk stratification by identifying and phenotyping subgroups of patients according to their physiological response to inflammation.

Multimodal phenotyping that integrates intraoperative data has further expanded AI applications. In a large cohort, ML identified distinct phenotypes of patients characterized by severe impairment in metabolic and immunological responses, hemodynamic instability, higher vasopressor requirements, and higher rates of organ dysfunction post-CPB [78].

Beyond conventional inflammatory markers, AI-based predictive models could include biomarkers such as presepsin, soluble urokinase plasminogen activator receptor (suPAR), circulating cell-free DNA, microRNA, and proteomic or metabolomic signatures. Their integration may increase the potential for early risk stratification and improve clinical judgment and management by identifying patient subgroups with distinct risks and outcomes.

A further application of AI involves the real-time integration of extracorporeal circulation data by monitoring different parameters, such as perfusion data, flows, pressures, oxygen delivery and consumption, hemoglobin and hematocrit levels, and lactate levels, allowing a risk assessment for the intraoperative and early postoperative periods, with potential applications extending to other mechanical circulatory support devices [79].

However, current models remain limited by retrospective validation. Most performant models (XGBoost AUC > 0.92; GBM AUC > 0.85) have been validated on retrospective cohorts. Additional limitations include variable standardization and the lack of prospective real-time integration of inflammation biomarkers with CEC data. Further multicenter validation and interoperable platforms are required before AI can become a routine component of Smart ICU systems [75,76,77,78,79,80].

**Figure 1:**
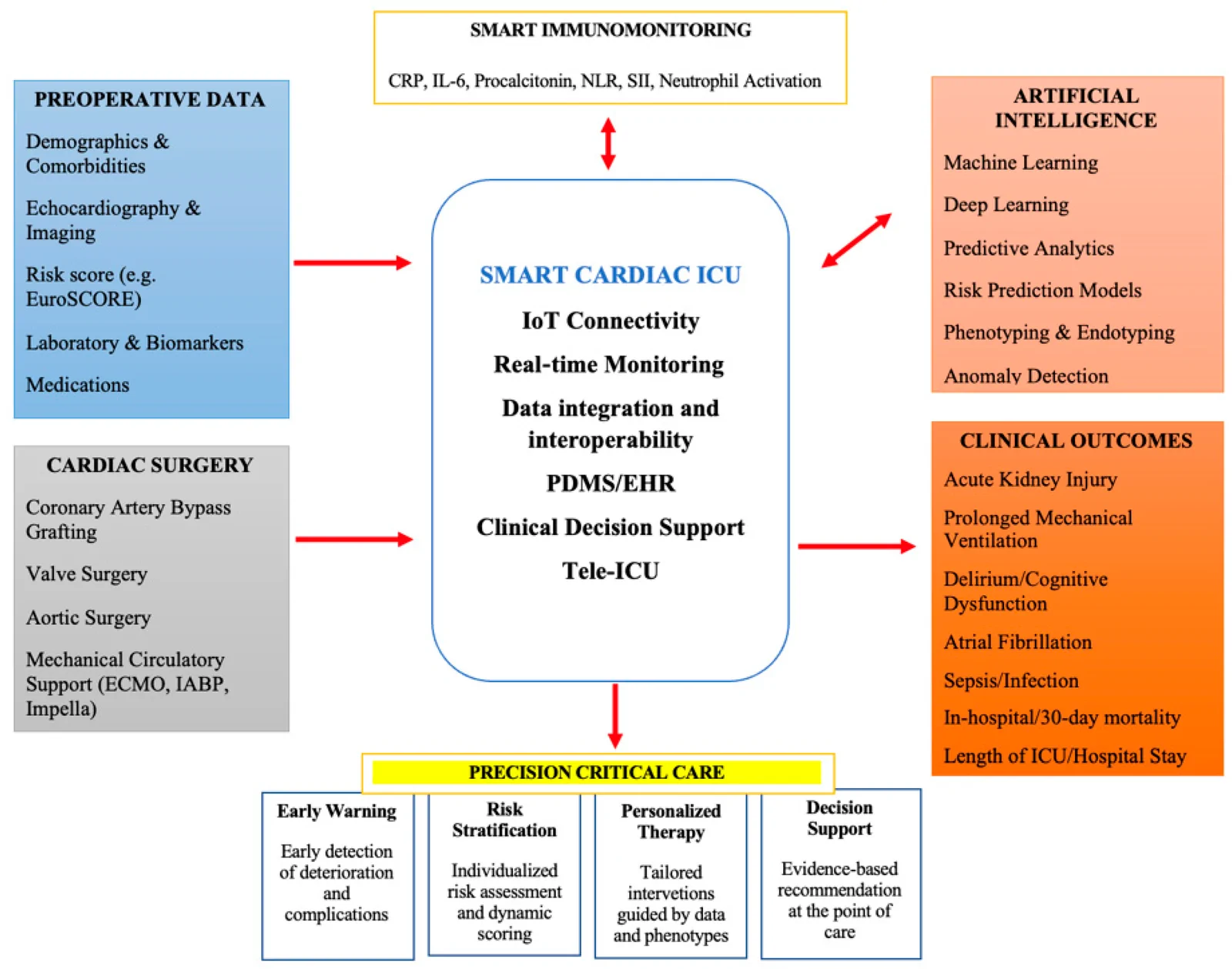
Smart Cardiac ICU Ecosystem: integration of perioperative data, immune monitoring, artificial intelligence, and clinical decision support for precision critical care. CPB—cardiopulmonary bypass; ECC—extracorporeal circulation; EHR—electronic health record; PDMS—patient data management system.

Figure 1 illustrates the proposed Smart Cardiac ICU data flow, in which perioperative and procedural data, immune-monitoring, predictive algorithms, and clinical decision support systems are integrated within a single digital infrastructure.

Table 5 contrasts the conventional ICU tools with Smart Cardiac ICU analytics.

## 4. Implementation Barriers, Governance, and Future Directions

### 4.1. From Concept to Clinical Practice

Implementation of this concept into clinical practice requires several conditions: data standardization, external validation, model explainability, efficient training of healthcare workers, cybersecurity, and cost-effectiveness. Digital infrastructure investment is required, while the costs of implementation and maintenance represent major barriers in current practice. In addition to prospective validation, further studies should evaluate the cost-effectiveness of AI-based technologies.

Table 6 summarizes the principal barriers currently encountered and required solutions.

### 4.2. Governance and Data Ethics

Governance must address confidentiality concerns, the risk of bias, and traceability of decisions. It also requires transparent and sustainable development, accountability for treating physicians, and continuous monitoring of results following implementation.

Cloud-based platforms carry an increased risk of cyberattacks and data breaches—robust cybersecurity is mandatory before clinical implementation of Smart ICUs. Furthermore, compliance with the General Data Protection Regulation (GDPR) is essential for protecting patient privacy and confidentiality. Fairness, transparency, and the risk of bias must be addressed to ensure safe implementation, and AI systems should comply with regulatory frameworks. The European AI Act classifies healthcare AI as a high-risk application, limited by the black-box concept, risk of bias, and the need for transparency. Similarly, the FDA emphasizes Good Machine Learning Practice, underscoring the need for technical performance and transparency, accountability, and prospective clinical validation for the clinical implementation of AI tools.

AI tools will not replace clinical judgment; therefore, responsibility remains in the hands of the clinicians. Continuous monitoring of system performance after implementing Smart ICU designs will be required [82]. Future research should focus on implementation studies and evaluating patient safety and system efficiency following the implementation in clinical practice of AI tools [83].

### 4.3. Future Directions

The future of Smart ICU lies in the transition from predictive to actionable systems that can be integrated into clinical practice. Current technologies focus on AI tools that address mechanical ventilation, measuring ventilatory parameters and predicting mechanical ventilation-associated adverse effects; alarm prioritization, reducing alarm fatigue and improving the reaction time to emergency situations; and postoperative risk assessment, using scoring systems and predictive factors that can influence postoperative outcomes. The FIRST-ICU model, which combines graph neural networks and LSTM to process data sequentially, plays a crucial role in the future direction of integrating AI into clinical practice, supporting AI-augmented decision-making [50].

A key future direction is the prospective, multicenter validation and standardization of AI assessment tools. The current literature is limited by the scarcity of prospective studies and lack of evidence regarding the clinical impacts of AI implementation in ICUs. A meta-analysis of 54 ML models for predicting postoperative cardiac complications in 927,113 patients reported better performance over traditional scores but was limited by inconsistencies in calibration methods and clinical integration [82].

Risk stratification and predictive scores should incorporate multimodal data integration, combining several parameters, such as physiological monitoring, laboratory data, biomarkers, and imaging. Hamza et al. proposed a neuroprotection framework in cardiac surgery, integrating imaging, inflammatory and metabolic biomarkers, neuromonitoring (TCD, NIRS, EEG/BIS), and ML models for predicting postoperative delirium and cognitive dysfunction [41]. In the cardiac surgical ICU, implementation of ML models could provide a better understanding of inflammation trends and complications, enabling “smart immune-monitoring”. Early identification of inflammatory phenotypes could support personalized perioperative immunomodulatory strategies in the future.

Future Smart Cardiac ICUs have the potential to evolve beyond predictive analytics. The concept of a digital twin is evolving, creating a virtual representation of a patient that continuously integrates clinical, physiological, laboratory, and imaging data, used to simulate disease progression, predict treatment responses, and support personalized decision-making [83]. Genomic and molecular data can be combined with laboratory biomarkers to characterize specific responses to surgery and inflammatory syndrome, while autonomous monitoring systems may further enhance patient management by extending monitoring of the patient outside the ICU environment. Deep learning-based multimodal data fusion has the potential to improve the accuracy of AI models, with great potential for diagnostic and predictive models [84]. Standardized frameworks are essential for the validation of AI models, and further prospective validation studies are mandatory before clinical implementation occurs.

The current AI models that have been developed and internally validated have shown promising performance, but their use is limited by the lack of prospective and external validation. Future research should focus on external validation, model calibration, and transportability—areas in which current models still encounter challenges. Prospective multicenter studies and randomized implementation trials are needed before widespread clinical implementation so as to evaluate real-world performance and clinical impact.

Successful adoption of AI-based technologies requires no disruption in clinical workflows as well as acceptance by clinicians, and, as mentioned previously, investment in digital infrastructure and cost-effectiveness demonstrations. Beyond transparency, AI models require regulatory approval, robust cybersecurity, and appropriate data governance to ensure safe deployment of these technologies into clinical practice. Future research should address these limitations before widespread implementation of AI platforms occurs.

Lastly, the future of Smart ICU will also be shaped by an interdisciplinary collaboration among clinicians, informaticians and statisticians in order to achieve safe, transparent, and sustainable Smart ICU development and implementation in clinical practice [79].

## 5. Limitations

This narrative review has several limitations. First, the literature selection was not exhaustive; rather than following a predefined, reproducible search protocol, studies were identified and included on the basis of their conceptual relevance to the Smart Cardiac ICU framework, which introduces the possibility of selection bias and means that some pertinent reports may not have been captured. Second, no formal assessment of risk of bias or methodological quality was performed for the included studies; therefore, the individual findings summarized here should be interpreted with caution and are not weighted according to their evidentiary strength. Third, the objective of this review is deliberately conceptual and translational rather than confirmatory: it aims to integrate heterogeneous evidence into a coherent architectural and mechanistic model and to identify translational opportunities for perioperative inflammation monitoring rather than to provide pooled effect estimates or definitive clinical recommendations. Accordingly, the conclusions should be regarded as hypothesis-generating and require prospective, multicenter validation before clinical implementation.

## 6. Conclusions

The Smart Cardiac ICU has the potential to transform intensive care units from device-centered environments into an integrated ecosystem, combining continuous monitoring, digital integration, predictive analytics, decision support, and telemedicine. While the benefits are promising—early detection of specific changes in clinical behavior, enhanced workflows and patient-centered care, real-time data analysis, and prediction of critical conditions—these approaches require clinical validation through prospective future research.

Future research should focus on developing standardized, multicenter datasets. Prospective randomized studies are mandatory, along with external validation studies of existing AI models. Multimodal data fusion and explainable AI have the potential to improve model generalizability and interoperability, supporting a transition from artificial intelligence to augmented clinical intelligence.

This review links the Smart Cardiac ICU concept to perioperative inflammation in cardiac surgery involving extracorporeal circulation and AI-based inflammatory phenotyping. We propose the cardiac surgical ICU as a model for data-driven monitoring of inflammatory and immunological changes, integrating the kinetics of specific inflammatory biomarkers with procedural data to support clinical decision-making. Post-CPB inflammatory syndrome may serve as a demonstrative model for data-driven critical care and provide future directions for Smart ICU models.

## Figures and Tables

**Table 1:** Related concepts in digital critical care.

Concept	Core Definition	Primary Function
Digital ICU Intelligent ICU Tele-ICU	An ICU that uses digital technology to continuously monitor, store, and analyze data	Digitalization and documentation of data
	A digital ICU that uses machine learning models in order to generate predictive algorithms	Analysis and prediction from collected data
	A remote central hub used for monitoring and supporting distant ICUs	Extend access to critical care expertise
Smart ICU concept	A unified ecosystem collecting heterogeneous data and creating interoperable, analytic digital support for clinical decision-making	Integration of the above into a unified, interoperable system
	**Core definition**	**Primary function**
Digital ICU Intelligent ICU Tele-ICU Smart ICU	An ICU that uses digital technology to continuously monitor, store, and analyze data	Digitalization and documentation of data
	A digital ICU that uses machine learning models in order to generate predictive algorithms	Analysis and prediction from collected data
	A remote central hub used for monitoring and supporting distant ICUs	Extend access to critical care expertise
	A unified ecosystem collecting heterogeneous data and creating interoperable, analytic digital support for clinical decision-making	Integration of the above into a unified, interoperable system
Digital ICU concept	**Core definition**	**Primary function**
	An ICU that uses digital technology to continuously monitor, store, and analyze data	Digitalization and documentation of data

**Table 2:** Data sources in the Smart Cardiac ICU and their predictive utility.

Data Source	Variables	Potential Predictive Utility	Data Source
Hemodynamic monitoring Mechanical ventilation Cardiopulmonary bypass and other ECC	Heart rate, central venous pressure, cardiac output, invasive/non-invasive arterial pressure	Hemodynamic instability, low cardiac output, vasoplegia	Hemodynamic monitoring
	Tidal volume, PEEP, pressure support, driving pressure, compliance	Prolonged ventilation, weaning parameters, ventilation-associated complications	Mechanical ventilation
	Pump flow, pressures, oxygen delivery and consumption, bypass and ischemia time	Inflammatory response, organ dysfunction requiring organ support therapies	Cardiopulmonary bypass and other ECC
Laboratory parameters Inflammatory biomarkers	Lactate, creatinine, hemoglobin, hematocrit, platelets, arterial and venous oxygen saturation, PaO_2_/FiO_2_	AKI, hypoperfusion, transfusion requirements, cardiac output, lung function	Laboratory parameters
	CRP, IL-6, neutrophil activation, NLR, SII	SIRS, prolonged ventilation, AKI, atrial fibrillation, low cardiac output syndrome, organ support therapy need, mortality	Inflammatory biomarkers
Clinical scores Medication and fluids Vasopressors and inotropes Urine output	EuroSCORE II, STS score, SOFA	Baseline and evolving risk stratification	Clinical scores
	Doses, pump infusion rates, fluid balance	Fluid overload, dosing safety, renal function	Medication and fluids
	Type, dose, duration	Vasoplegia, hemodynamic instability, low cardiac output syndrome, prolonged ICU stay, mortality	Vasopressors and inotropes
	Hourly measurement of urine output, fluid balance	AKI, need for continuous renal replacement therapy	Urine output
Data source Hemodynamic monitoring	Variables	Potential predictive utility	Data source
	Heart rate, central venous pressure, cardiac output, invasive/non-invasive arterial pressure	Hemodynamic instability, low cardiac output, vasoplegia	Hemodynamic monitoring

AKI—acute kidney injury; CRP—C-reactive protein; ECC—extracorporeal circulation; EuroSCORE II—European System for Cardiac Operative Risk Evaluation II; IL-6—interleukin-6; NLR—neutrophil-to-lymphocyte ratio; SII—systemic immune-inflammation index; SIRS—systemic inflammatory response syndrome; SOFA—Sequential Organ Failure Assessment; STS—Society of Thoracic Surgeons.

**Table 3:** Summary of current evidence regarding AI models, validation, and outcomes.

Study	Study Type	Sample Size	AI Model/Digital Technology	Validation	Clinical Applications	Main Findings
Van de Sande et al. [1]	Systematic review	494 studies	AI models in ICU		Clinical readiness of AI in ICU	89.3% at readiness level ≤ 4; none in routine practice; 80.9% at high risk of bias
Berkhout et al. [7]	Systematic review	1263 studies	AI models in ICU	24% externally validated	Operationalization of AI in ICU	74% at TRL ≤ 4; only 2% at TRL ≥ 6; 53% high risk of bias
Kong et al. [35]	Retrospective, single-center MIMIC III database	16,688 Sepsis 3 adults	GMB, random forest	No external validation	In-hospital mortality for sepsis patients	GBM AUROC 0.845 vs. SAPS II 0.770
Zepeda-Lugo et al. [36]	Systematic review and meta-analysis	33 studies	ML	External validation in 9.9%	Prolonged ICU stay	AUROC 0.9005 (0.8890–0.9121) from 10 studies; 75.8% high risk of bias
Xue et al. [38]	Retrospective, single-center	111,888	GBT, random forest, LR, SHAP	No external validation	AKI, delirium, DVT, pulmonary embolism, pneumonia	GBT best for 4/5 outcomes (pneumonia 0.905, AKI 0.848, delirium 0.762)
Sinha et al. [39]	Retrospective analysis	227,087 cardiac surgery patients	XGBoost, random forest, LR	No external cohort	In-hospital mortality after cardiac surgery	XGBoost AUC 0.834 vs. EuroSCORE II 0.818
Bahram et al. [40]	Retrospective, single-center	1098 cardiac surgery patients	XGBoost, LDA	No external validation	Postoperative mechanical ventilation requirement	Accuracy 0.850, sensitivity 0.859, specificity 0.841;
Squiccimarro et al. [48]	Retrospective single-center	1908 cardiac surgery patients	ML	No external validation	Post-CPB SIRS and 30-day mortality	SIRS in 28.7%; mortality 12.2% vs. 1.5%
Kong et al. [49]	Retrospective three-centers	10,847 procedures; 1006 features	GMB	Three independent external datasets	Five phenotypes	Phenotype E showed AKI 66.9%, acute liver failure 38.8%, and mortality 11.4%
Aikodon et al. [50]	Retrospective	MIMIC-IV database − n = 23,926	CNN baselines	Independent external validation	Need for ICU interventions	AUC-ROC > 0.98 with Brier < 0.035 internally, falling to >0.90 externally

AI—artificial intelligence; AKI—acute kidney injury; AUC/AUROC—area under the receiver operating characteristic curve; CBP—cardiopulmonary bypass; CNN—convolutional neural network; DL-GMB—gradient boosting machine; GBT—gradient boosting trees; ICU—intensive care unit; LDA—linear discriminant analysis; LR—logistic regression; ML—machine learning; SIRS—systemic inflammatory response syndrome; TLR—technology readiness level.

**Table 4:** Performance metrics of current AI tools in comparison to conventional scores.

Study	Objective	AI Model	Conventional Score	Performance of AI Model	Performance of Comparator	Validation
Kong et al. [35]	In-hospital mortality in sepsis	GMB, random forest	SAPS II	GBM: AUROC 0.845 (0.837–0.853); sensitivity 0.771; specificity 0.755; random forest 0.829	SAPS II: AUROC 0.770 (0.760–0.780); sensitivity 0.697; specificity 0.714;	Single database, no external validation
Zepeda-Lugo et al. [36]	Prolonged ICU length of stay	ML/DL		AUROC 0.9005 (0.8890–0.9121)	-	True external validation in 9.9%;
Xue et al. [38]	Postoperative complications after surgery	GBT, random forest, LR, SHAP	none	GBT: pneumonia AUROC 0.905 (0.903–0.907); DVT 0.881 (0.878–0.884); AKI 0.848 (0.846–0.851); delirium 0.762 (0.759–0.765)	-	Single-center study; no external validation
Sinha et al. [39]	In-hospital mortality after cardiac surgery	XGBoost, random forest, LR	EuroSCORE II	XGBoost: AUC 0.834 (0.834–0.834); Brier 0.0236; random forest AUC 0.834	EuroSCORE II: AUC 0.818	No external cohort

**Table 5:** Conventional ICU tools versus Smart Cardiac ICU analytics based on literature review.

Domain	Conventional Tool	Possible Model	Outcome Targeted	Current Limitation
Mortality	APACHE, SOFA, SAPS II	RF, XGBoost, LSTM-RNN [34,35,36]	ICU mortality	Retrospective design, with limited external validation
Length of stay	-	Meta-analysis with AUROC ≈ 0.90 [36]	ICU/hospital LOS	Heterogeneous definitions
Perioperative risk	EuroSCORE II, STS score	MySurgeryRisk, POTTER, GBT [37,38]	Morbidity, mortality, postoperative AKI, and pneumonia	Calibration and integration variable
Tele-ICU	Telemonitoring	Integrated analytics platforms [61,81]	LOS, ventilation, vasopressor use	RCT evidence mixed (TELESCOPE neutral)
Perioperative inflammation	CRP and other biomarkers	RF, phenotyping [48,77]	SIRS, postoperative AKI, organ dysfunction	Retrospective design, no real-time integration

AKI—acute kidney injury; APACHE—Acute Physiology and Chronic Health Evaluation; AUROC—area under the receiver operating characteristic curve; CPB—cardiopulmonary bypass; CRP—C-reactive protein; ECC—extracorporeal circulation; GBT—gradient boosting trees; LOS—length of stay; LSTM-RNN—long short-term memory recurrent neural network; RF—random forest; SAPS II—Simplified Acute Physiology Score II; SIRS—systemic inflammatory response syndrome; SOFA—Sequential Organ Failure Assessment; STS—Society of Thoracic Surgeons; XGBoost—extreme gradient boosting.

**Table 6:** Barriers to Smart Cardiac ICU implementation and proposed solutions.

Barrier Category	Main Barrier	Proposed Solutions
Technical	Lack of interoperability, heterogeneous data	Common standards, proper data integration
Clinical	Retrospective studies, workflow disruption	Prospective validation; integration into existing workflow
Organizational	Infrastructure cost, resistance to change	Training; command center models
Ethical and legal concerns	Data privacy, risk of bias, accountability	Governance frameworks; clear responsibility

## Data Availability

No new data were created or analyzed in this study. Data sharing is not applicable to this article.

## Abbreviations

The following abbreviations are used in this manuscript:

AI	Artificial intelligence
AKI	Acute kidney injury
APACHE	Acute Physiology and Chronic Health Evaluation
AUC	Area under the curve
AUROC	Area under the receiver operating characteristic curve
BIS	Bispectral index
CDSS	Clinical decision support system
CPB	Cardiopulmonary bypass
CRP	C-reactive protein
DICOM	Digital Imaging and Communications in Medicine
ECC	Extracorporeal circulation
ECMO	Extracorporeal membrane oxygenation
EEG	Electroencephalography
EHR	Electronic health record
FHIR	Fast Healthcare Interoperability Resources
GBT	Gradient boosting trees
HL7	Health Level Seven
ICU	Intensive care unit
IoMT	Internet of Medical Things
IoT	Internet of Things
LIME	Local Interpretable Model-agnostic Explanations
LOS	Length of stay
LSTM	Long short-term memory
ML	Machine learning
NIRS	Near-infrared spectroscopy
NLR	Neutrophil-to-lymphocyte ratio
PDMS	Patient data management system
qSOFA	Quick SOFA
RF	Random forest
RNN	Recurrent neural network
SAPS II	Simplified Acute Physiology Score II
SHAP	SHapley Additive exPlanations
SII	Systemic immune-inflammation index
SIRS	Systemic inflammatory response syndrome
SOFA	Sequential Organ Failure Assessment
STS	Society of Thoracic Surgeons
TCD	Transcranial Doppler
TRL	Technology readiness level
XGBoost	extreme gradient boosting
